# Supplementary material for: Ongoing Failure to Deliver Guideline-Concordant Care for Patients with Pancreatic Cancer
Source: Cancers (Basel). 2025 Jan 7;17(2):170. doi: 10.3390/cancers17020170 (PMC11763659; doi:10.3390/cancers17020170)
Supplement: Supplementary file 1 [file cancers-17-00170-s001.zip › cancers-3400447-supplementary.pdf]

**Supplementary Materials**:

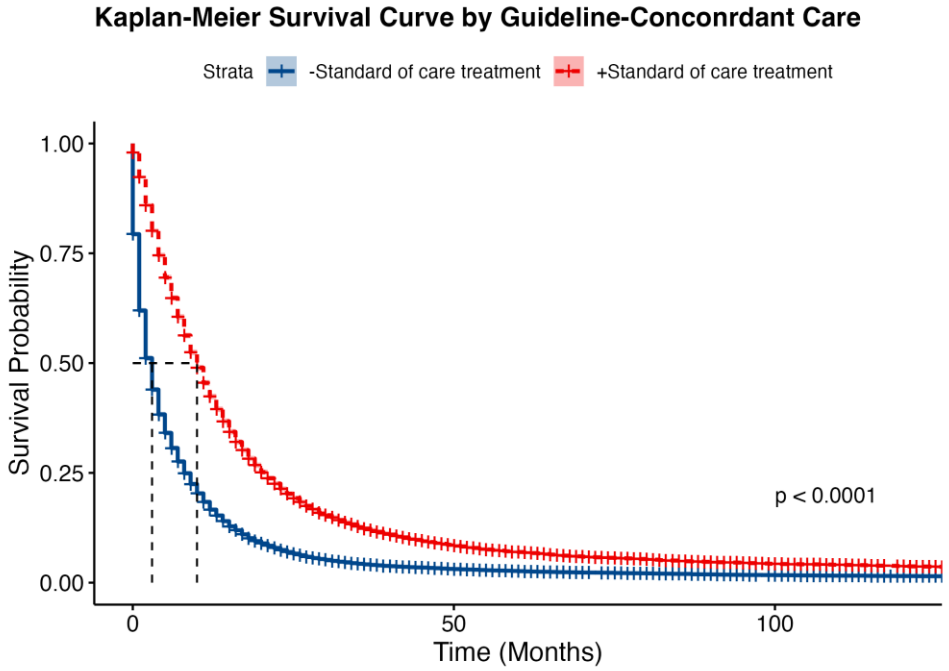


**Supplementary Figure S1**. Kaplan–Meier survival curves for patients receiving GCC. This Kaplan–Meier survival analysis compares the survival probabilities over time for pancreatic cancer patients based on whether they received GCC. Patients who received care adhering to guidelines (red line) consistently demonstrated higher survival probabilities compared to those who did not (blue line).

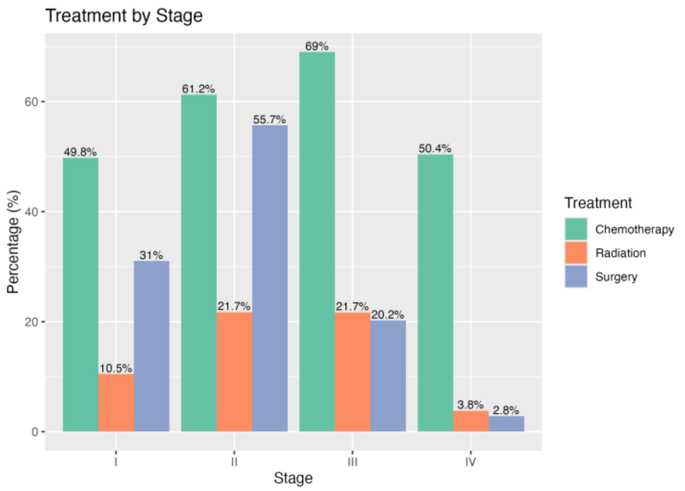


**Supplementary Figure S2**. Stage-specific treatment patterns for patients with PDAC. The predominant treatment for patients with stage 1 or 2 PDAC was chemotherapy followed by surgery, while chemotherapy followed by radiotherapy was more commonly administered to those with stage 3 and 4 disease.

**Supplementary Table S1**. Cox proportional hazards analysis of nature of treatment's association with overall survival in patients with stage 1 PDAC.

| Variable | HR 1 | 95% CI 2 | p-Value |
| --- | --- | --- | --- |
| Age | 1.02 | 1.01–1.02 | <0.0001 |
| Sex(female) | 0.94 | 0.88–1.01 | 0.092 |
| Race | | | |
| White | — | — | |
| Asian | 0.92 | 0.82–1.03 | 0.14 |
| Black | 1.07 | 0.94–1.23 | 0.31 |
| Hispanic | 1.00 | 0.91–1.11 | 0.97 |
| Other | 0.93 | 0.57–1.52 | 0.77 |
| Insurance | | | |
| Private | — | — | |
| Medicare | 1.02 | 0.94–1.10 | 0.71 |
| No Insurance | 1.03 | 0.72–1.48 | 0.86 |
| Other | 1.00 | 0.80–1.24 | 0.97 |
| Location of Residence | | | |
| Metropolitan | — | — | |
| Micropolitan | 1.02 | 0.85–1.23 | 0.79 |
| Small Town | 1.52 | 1.07–2.16 | 0.018 |
| Rural Areas | 1.05 | 0.76–1.45 | 0.77 |
| CD Index | | | |
| 0 | — | — | |
| 1 | 1.16 | 1.06–1.27 | 0.0020 |
| >2 | 1.37 | 1.26–1.49 | <0.0001 |
| Surgery | | | |
| no | — | — | |
| yes | 0.22 | 0.20–0.25 | <0.0001 |
| Chemotherapy | | | |
| no | — | — | |
| yes | 0.67 | 0.62–0.72 | <0.0001 |
| Radiation | | | |
| no | — | — | |
| yes | 0.91 | 0.82–1.01 | 0.089 |

1 HR = Hazard Ratio; 2 CI = Confidence Interval; CD Index = Charlson–Deyo Comorbidity Index.

**Supplementary Table S2**. Multivariable Analysis of factors associated with not having surgery in the initial treatment plan in patients with stage 1 PDAC.

| Variable | OR 1 | 95% CI 1 | p-Value |
| --- | --- | --- | --- |
| Age | 1.04 | 1.03–1.05 | <0.001 |
| Sex (female) | 0.88 | 0.78–1.00 | 0.057 |
| Race | | | |
| White | — | — | |
| Asian | 0.93 | 0.76–1.13 | 0.45 |
| Black | 1.46 | 1.14–1.89 | 0.003 |
| Hispanic | 1.05 | 0.88–1.26 | 0.60 |
| Other | 1.05 | 0.44–2.53 | 0.92 |
| Insurance | | | |
| Private | — | — | |
| Medicare | 1.08 | 0.94–1.26 | 0.27 |
| No Insurance | 1.64 | 0.83–3.33 | 0.16 |
| Other | 1.02 | 0.70–1.50 | 0.91 |

| | | | |
|---|---|---|---|
| CD Index | | | |
| 0 | — | — | |
| 1 | 1.26 | 1.07–1.49 | 0.007 |
| >2 | 1.43 | 1.23–1.66 | <0.001 |
| Location of Residence | | | |
| Metropolitan | — | — | |
| Micropolitan | 0.90 | 0.65–1.24 | 0.51 |
| Small Town | 1.30 | 0.66–2.64 | 0.46 |
| Rural Areas | 1.00 | 0.56–1.82 | >0.99 |

1 Or = Odds Ratio; CI = Confidence Interval; CD Index = Charlson–Deyo Comorbidity Index.

**Supplementary Table S3**. Multivariable analysis of factors associated with refusing surgery in patients with Stage 1 PDAC.

| Variable | OR1 | 95% CI1 | p-Value |
|---|---|---|---|
| Age | 1.06 | 1.04–1.08 | <0.001 |
| Sex (female) | 1.21 | 0.89–1.65 | 0.23 |
| Race | | | |
| White | — | — | |
| Asian | 0.86 | 0.51–1.37 | 0.55 |
| Black | 0.73 | 0.34–1.40 | 0.39 |
| Hispanic | 1.12 | 0.71–1.69 | 0.62 |
| Other | 0.00 | | 0.99 |
| Insurance | | | |
| Private | — | — | |
| Medicare | 1.13 | 0.79–1.66 | 0.52 |
| No Insurance | 1.93 | 0.30–6.85 | 0.39 |
| Other | 1.11 | 0.33–2.87 | 0.84 |
| CD Index | | | |
| 0 | — | — | |
| 1 | 0.84 | 0.56–1.26 | 0.41 |
| >2 | 0.84 | 0.59–1.20 | 0.33 |
| Location of Residence | | | |
| Metropolitan | — | — | |
| Micropolitan | 0.28 | 0.05–0.91 | 0.08 |
| Small Town | 0.00 | 0.00–1,048,484 | 0.98 |
| Rural Areas | 0.39 | 0.02–1.85 | 0.36 |

1 Or = Odds Ratio; CI = Confidence Interval; CD Index = Charlson–Deyo Comorbidity Index.
